# 1406. A Systematic Quality Improvement Initiative to Improve Chagas Disease Screening in Patients with HIV at a Large Safety Net Clinic in Denver

**DOI:** 10.1093/ofid/ofad500.1243

**Published:** 2023-11-27

**Authors:** Lilian Vargas Barahona, Andrés F Henao Martínez, Margaret P McLees, Edward Gardner, Kellie Hawkins

**Affiliations:** University of Colorado, Omaha, Nebraska; University of Colorado Anschutz Medical Campus, Aurora, CO; Public Health Institute at Denver Health, Denver Health and Hospital Authority, Denver, Colorado; Denver Health and Hospital Authority, Denver, Colorado; Denver Health Medical Center, Denver, Colorado

## Abstract

**Background:**

Chagas Disease (CD) is a neglected parasitic disease that affects people living in continental Latin America. Immigrants from these countries or those born to mothers from Latin America are at risk of developing disease. Detection of cases before end-organ damage has occurred is crucial for treatment to be beneficial. Patients with HIV (PWH) may be at high risk of reactivation and death. We sought to screen PWH at risk of CD that receive medical care in the safety net hospital for the Denver area.

**Methods:**

We identified 325 PWH that receive care in the Denver Health system who self-identified as born in Latin American countries. We ordered serological Trypanosoma cruzi testing for all patients (T cruzi IgG, ELISA, ARUP Laboratories, UT). Providers were educated about the intervention and CD via email **(Figure 1)**. Concurrently, the Denver Health refugee clinic began a process to screen for CD among people at risk who do not have HIV.

**Figure 1.**
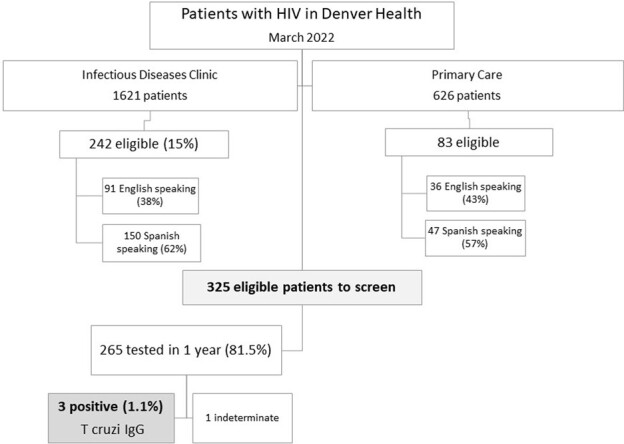
Chagas disease screening process in patients with HIV in Denver Health, March 2022 - March 2023

**Results:**

Between March 2021 and March 2022, a total of 265 patients completed testing (81.5%). 89.8% were male and the mean age was 45.4 years (SD +/- 11.5). Most patients had controlled HIV (mean CD4 624 +/- 303.5 cells/µL, HIV viral load < 30 cps/mL in 92%). Most patients were born in Mexico (80%), followed by El Salvador (5%). Three patients had a positive test, yielding a prevalence of 1.1%. One of these patients had an already confirmed and treated infection. The other 2 patients had uncontrolled HIV with CD4 count < 200 cells and have been lost to follow-up with inability to obtain confirmatory testing. Using a 2.2% prevalence of serologic positivity for CD in Hispanic patients, 90 patients at risk will need to be screened in order to detect a positive patient. Due to increased awareness of CD in our primary care clinics, a parallel 3-fold increase in testing in non-HIV patients was seen in the study period.

**Conclusion:**

Chagas disease screening in patients at risk can be performed in clinics that provide care for patients with HIV. Obtaining a second confirmatory test is challenging and processes to facilitate blood collection should be implemented to not delay testing. Increased awareness of medical providers leads to increased screening for this disease.

**Disclosures:**

**Edward Gardner, MD**, Cepheid: Grant/Research Support|Gilead Sciences: Grant/Research Support|ViiV Healthcare: Grant/Research Support

